# Refining Risk Criteria May Substantially Reduce Unnecessary Additional Surgeries after Local Resection of T1 Colorectal Cancer

**DOI:** 10.3390/cancers16132321

**Published:** 2024-06-25

**Authors:** Fernando Martínez de Juan, Samuel Navarro, Isidro Machado

**Affiliations:** 1 Unit of Gastroenterology and Digestive Endoscopy, Instituto Valenciano de Oncología, 46009 Valencia, Spain; 2 Department of Pathology, Universidad de Valencia, 46010 Valencia, Spain; 3 Centro de Investigación Biomédica en Red de Cáncer (CIBERONC), 46009 Valencia, Spain; 4 Department of Pathology, Instituto Valenciano de Oncología, 46009 Valencia, Spain; 5 Patologika Laboratory, Hospital Quirón-Salud, 46010 Valencia, Spain

**Keywords:** T1 colorectal cancer, tumor budding, lymphovascular invasion, poorly differentiated clusters, differentiation grade, histologic grade, invasion depth, lymph node metastases

## Abstract

**Simple Summary:**

Current clinical practice guidelines support additional locoregional surgery after local resection of early colorectal cancer when one or more of the following risk factors are present: poor differentiation grade, lymphovascular invasion, deep submucosal invasion, moderate or high tumor budding, and deep margin involvement. The absence of all these risk criteria selects a subgroup of patients with very good prognosis that can safely avoid additional surgery. However, lymph node metastases are only identified in 10–20% of patients who ultimately undergo additional surgery, so that 80–90% of the surgeries turn out to be unnecessary. In this investigation, we find that indicating additional surgery in early colorectal cancer only when either moderate or high tumor budding, poorly differentiated clusters, or lymphovascular invasion are present could reduce unnecessary surgeries by 40–45% without significantly increasing the proportion of patients with lymph node metastases who are not offered additional surgery.

**Abstract:**

Background: The low positive predictive value for lymph node metastases (LNM) of common practice risk criteria (CPRC) in T1 colorectal carcinoma (CRC) leads to manyunnecessary additional surgeries following local resection. This study aimed to identify criteria that may improve on the CPRC. Methods: Logistic regression analysis was performed to determine the association of diverse variables with LNM or ‘poor outcome’ (LNM and/or distant metastases and/or recurrence) in a single center T1 CRC cohort. The diagnostic capacity of the set of variables obtained was compared with that of the CPRC. Results: The study comprised 161 cases. Poorly differentiated clusters (PDC) and tumor budding grade > 1 (TB > 1) were the only independent variables associated with LNM. The area under the curve (AUC) for these criteria was 0.808 (CI 95% 0.717–0.880) compared to 0.582 (CI 95% 0.479–0.680) for CPRC. TB > 1 and lymphovascular invasion (LVI) were independently associated with ‘poor outcome’, with an AUC of 0.801 (CI 95% 0.731–0.859), while the AUC for CPRC was 0.691 (CI 95% 0.603–0.752). TB > 1, combined either with PDC or LVI, would reduce false positives between 41.5% and 45% without significantly increasing false negatives. Conclusions: Indicating additional surgery in T1 CRC only when either TB > 1, PDC, or LVI are present could reduce unnecessary surgeries significantly.

## 1. Introduction

**Figure 1:**
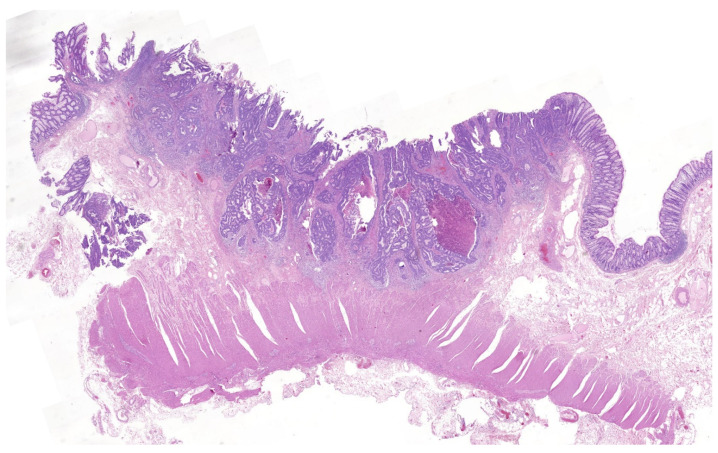
T1 colorectal cancer with deep submucosal invasion. H&E ×1.4.

The corner stone of colorectal cancer treatment is tumor resection and must include locoregional lymph node resection when lymph node metastases are known or suspected, or where the probability is high, as is the case for adenocarcinomas involving muscularis propria or deeper bowel layers. According to a recent metanalysis, when tumor infiltration is limited to the submucosa (T1), the risk of lymph node metastases is 11.2% [1]. Thus, in most of these cases, local resection (LR) would be curative, and few patients would benefit from regional lymph node resection if locoregional resection was carried out in all T1 CRC. Due to the lack of a non-invasive methods able to reliably identify lymph node metastasis (and so the group of patients who would really benefit from locoregional resection), the indication for additional surgery after local resection (mostly endoscopic) of a T1 CRC relies on the probabilities of lymph node metastases in association with adverse histopathological features. Current clinical practice guidelines suggest individual decision-making [1,2,3,4] but support additional locoregional surgery (ALRS) when one or more of the following risk factors are found in the local resection specimen: poor differentiation grade, lymphovascular invasion (LVI), deep submucosal invasion (Figure 1), moderate or high tumor budding (TB), and deep margin involvement. The absence of all of these common practice risk criteria (CPRC) has a good negative predictive value that reliably selects a subgroup of patients with very good prognosis that can safely avoid additional surgery. However, the positive predictive value of some of these risk factors, such as depth of invasion, is suboptimal and can lead to a high proportion of unnecessary surgeries. The result is that LNM are found in only 10–20% of cases that undergo additional surgical resection after an initial local T1 CRC resection [5,6,7,8]. This means that 80–90% of these patients are exposed to the risks and sequelae of major surgery without any benefit other than confirming the otherwise unlikely absence of regional nodal tumor spread. The proven efficacy of CRC screening programs has led to their implementation worldwide, with a continuously growing number of colonoscopies together with the endoscopic resection of lesions that finally turn out to be T1 CRC. As a result, clinicians and patients are frequently faced with the dilemma of accepting additional surgery with limited benefits in most cases, or rejecting surgery and relying solely on follow-up, assuming a low, but not null, risk of LNM when left untreated. In this scenario, refinement of the current criteria, novel risk factors, or new strategies are needed to lower the proportion of unnecessary surgeries without worsening the oncological outcomes. In this paper, we evaluate the current risk factors used in daily clinical practice, and some others, such as poorly differentiated clusters (PDC), described elsewhere [5,6,7,8,9] but not included in current practice guidelines. PDC are defined as groups of five or more tumor cells without gland formation, which correlate with prognosis, risk of lymph node involvement, and distant metastases in CRC [10,11,12,13,14,15,16,17,18]. As with TB, PDC are an expression of epithelial–mesenchymal transition (EMT) and may be easier to identify than TB because of their larger size. Based on the results of our study, we present three criteria, TB > 1, PDC, and LVI, that may improve ALRS decision-making following LR.

## 2. Materials and Methods

We studied the different prognostic factors, mainly histological, in all the T1 CRC cases treated at the Instituto Valenciano de Oncología (IVO) between 1 January 1993 and 30 August 2017. Exclusion criteria were patients with CRC stages other than T1, classic familial adenomatous polyposis phenotype, or any other polyposis phenotype with diagnosed pathogenic mutation, previous history of CRC, and patients with other forms of cancer and with metastases in whom the origin of metastases could not be determined.

Our study comprised two analyses. In the first, a logistic regression analysis was performed to determine the association of specific clinical and pathological parameters with LNM, as this is the most frequently used outcome in the evaluation of risk of T1 CRC disease. Only those patients who had undergone ALRS or primary locoregional surgery (PLRS) could be included in this analysis. A second analysis was then carried out to avoid the loss of potentially relevant information from the cases that were followed up after LR and did not undergo ALRS, considering together all adverse oncological outcomes (not only LNM, but also synchronous or metachronous metastases or local recurrence) as a single variable (‘poor outcome’). Thus, patients with LR alone and with at least 2 years’ follow-up were also included in this analysis. Receiver Operating Characteristic curves (ROC curves) were calculated for the resulting criteria and the Akaike information criterion (AIC) was used to select the best performers. The diagnostic capacity of the criteria obtained was compared with that of the CPRC.

When it was not possible to assess the depth of invasion or margin status due to fragmentation of the resected specimen, deep invasion and affected margin were assumed, as is common in daily practice. All statistical calculations were performed using the statistical software packages MedCalc [19] and R [20].

A minimum sample size of 142 cases was calculated based on the diagnostic performance of the studied variables, defined by the area under the curve (AUC) of the ROC curves. We assumed a type I error of 0.05, a type II error of 0.2, and a V Cramer correlation coefficient between histological grade and PDC of 0.4 in positive cases and 0.2 in negative cases. In addition, we assumed an expected prevalence of LNM of 11.2% and a hypothetical AUC of 0.57 for differentiation grade and 0.83 for PDC in preliminary observations at our institution.

All the slides were scanned and reviewed by a pathologist with more than 20 years’ experience in digestive pathology (I.M.).

Each treatment decision had been made on an individual basis by a multidisciplinary committee, relying primarily on conventional risk factors and the specific clinical situation of each patient. The follow-up of the patients was performed as clinically indicated.

The ethics committee of the IVO approved the study protocol on 29 March 2017. The committee also granted a waiver of informed consent from the participants since this is an observational study without intervention and no additional treatments or surgeries would be recommended to these participants based on the results.

## 3. Variables

Epidemiological variables, endoscopic resection-related variables, and pathological variables were studied. We provide some explanations about certain variables where there may be controversies in the way they are assessed, or where they are relatively new and/or not commonly used in routine clinical practice.

Histological grade was categorized as either low grade (G1 or well-differentiated tumors and G2 or moderately differentiated tumors) or high grade (G3 or poorly differentiated tumors and G4 or undifferentiated tumors). We defined tumor differentiation grade according to the percentage of glandular formation, rather than by the less differentiated component (i.e., CRC was classified as G1 when showing more than 95% of gland formation, G2 when gland formation ranged between 50% and 95%, G3 when gland formation was lower than 50%, and G4 in cases of less than 5%of gland formation).

Tumor budding was assessed by hematoxylin and eosin (H&E) staining and defined as a single tumor cell or a cell cluster of up to four tumor cells in one hotspot (in a field measuring 0.785 mm^2^) at the invasive front. The results were graded through a three-tier system (grade 1: 0–4; grade 2: 5–9; grade 3: 10 or more buds) following the current recommendations for reporting tumor budding in colorectal cancer [21]. As per histological grade, results were categorized as TB grade 1 (or low grade) and TB grade > 1 (moderate or high grade), as is usually considered in clinical decision-making.

**Figure 2:**
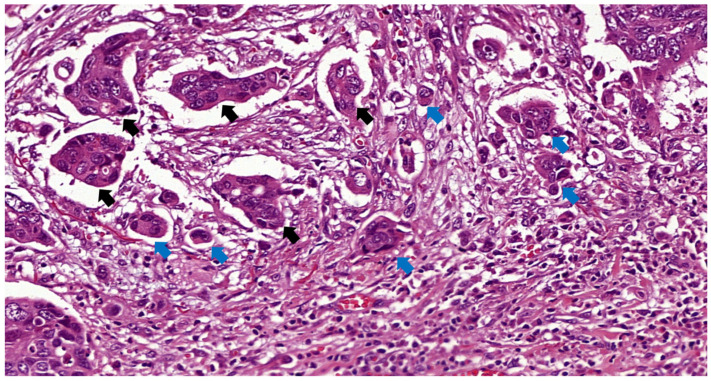
TB and PDC indicated by blue and black arrows, respectively, in a T1 rectal cancer H&E ×31.

PDC were defined according to Ueno et al. [16] as cancer clusters of at least five cancer cells and lacking a gland-like structure counted under a 20× lens (equivalent to a field of 0.785 mm^2^) in a field containing the highest number of clusters. PDC was regarded as a dichotomous variable (absent or present, unlike the three-tiered system used in TB), as most of the cases were endoscopic resections in which no grading is suggested due to the fewer number of clusters usually seen in endoscopic specimens compared to surgical pieces [18]. An example of a tumor with TB and PDC is shown in Figure 2.

A distance of 1 mm or less between the tumor and the resection surface was considered a positive deep margin, and negative when the distance was greater. In the case where PLRS was performed, the margin was always greater than 1 mm.

Depth of submucosal invasion was assessed according to JSCCR guidelines [1]. In short, the invasion depth was measured from the lower border of the muscularis mucosae MM or from the surface of the lesion when the MM could not be identified. A threshold of ≥1000 µm was used to define deep submucosal invasion in non-pedunculated lesions. In pedunculated lesions, the depth was measured from the neck of the polyp, with a threshold of ≥3000 µm for deep invasion. Depth of invasion was considered not assessable in piecemeal resections.

Width of submucosal invasion was measured as the widest distance between the horizontal limits of the submucosal invasion, parallel to the MM plane. As with depth of submucosal invasion, width was considered not assessable in piecemeal resections.

Background adenoma was defined as any remnant of adenoma component within the resected lesion.

Intra- and peritumoral inflammation were graded in two categories: absent or mild vs. moderate or intense.

**Figure 3:**
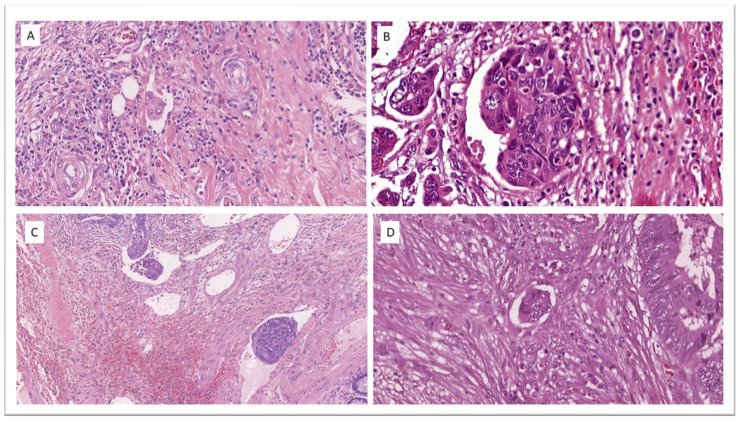
Lymphovascular invasion in four different cases of T1 colorectal cancer. (**A**) H&E ×32.4. (**B**) H&E ×41.4. (**C**) H&E ×13.4. (**D**) H&E ×33.0.

According to European recommendations [22], no additional staining other than H&E was used in the assessment of LVI (Figure 3) or any other variable.

## 4. Results

### Description of the Samples, Management, and Outcomes

**Figure 4:**
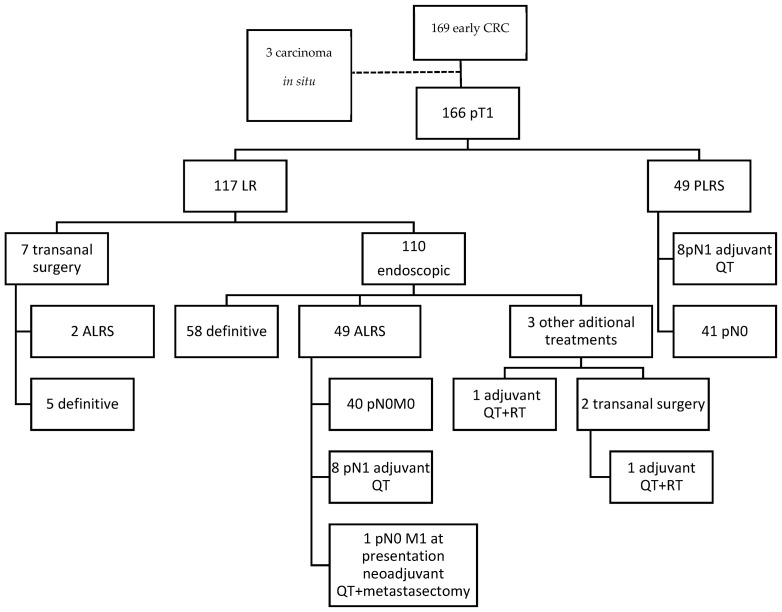
Flowchart of the clinical pathway followed by the patients included. ALRS: additional locoregional surgery; LR: local resection; PLRS: primary locoregional surgery; QT: chemotherapy; RT: radiotherapy.

A total of 169 T1 CRC diagnosed cases were retrieved (see Figure 4). Three were three excluded due to absence of an invasive component after review of the slides. Five more cases treated with LR alone were excluded from the poor outcome association analysis due to follow-up shorter than 2 years. Of the 166 included cases, 49 had initially been treated by PLRS, and 117 by LR (110 endoscopically and 7 through surgical transanal resection). ALRS was performed in 51 out of the 117 LR cases. In three of the rectal endoscopically treated cases, some form of additional treatment other than total mesorectal excision was performed: in two cases, LR was expanded through surgical transanal resection, followed by adjuvant treatment with chemotherapy and radiotherapy in one of these. In the third case, adjuvant treatment was administered directly after endoscopic resection. There was one case of synchronous liver metastasis. In 19 cases, ALRS was not performed despite being indicated as standard treatment: in 10 cases to preserve anal function, in 5 due to comorbidities, in 2 at the express wish of the patients, and in 2 due to a lack of identification of risk factors in the initial evaluation. LNM were found in 16 of the 100 patients who underwent locoregional surgery.

The median follow-up time was 71 months (0–289). Twenty-four patients were lost to follow-up. Seven losses occurred before the 2-year mark in the PLRS group. Another seven losses occurred between two and five years (three in the LR group, one in the group who underwent ALRS and three in the PLRS group). The median follow-up in these seven cases was 4 years and 2 months. The remaining 10 losses (5 in group 1, 3 in group 2, and 2 in group 3) occurred after five years, which is the minimum period usually considered for follow-up in colorectal cancer.

Six tumor recurrences were identified: two local, both after a transrectal local excision, and four distant. Two of the distant recurrences occurred in patients who had undergone endoscopic resection as the sole treatment, and the other two in patients who had undergone ALRS after LR. The average time until recurrence was 46.5 months (11–90 months). In three out of the six cases, conventional risk factors that had gone unnoticed in the initial evaluation were identified in the review of the slides for the study: LVI and TB grade 2 in one case treated with endoscopic resection only, TB grade 3 and deep submucosal invasion in one case of transrectal local excision, and deep submucosal invasion in another case of transrectal LR. Colorectal cancer was the cause of death in two of the patients with distant metastases, and in one of the patients with local recurrence who eventually developed distant metastases. Ten more patients died, four due to non-oncological causes and six due to cancers other than CRC.

## 5. Association with LNM

The association between the different risk factors and LNM was evaluated for the 100 patients who underwent locoregional surgery (49 PLRS and 51 ALRS) (Table 1).

The multivariate analysis included variables with statistical significance in the univariate analysis (age, rectal location, high histological grade, presence of lymphovascular invasion, TB grade > 1, and presence of PDC). Two different sets of risk criteria were obtained depending on the variable selection procedure used. With the backward method, only two variables were independently statistically significant: the presence of TB grade >1 and PDC (*TB* > 1 + *PDC*). Using the forward and stepwise methods, only the presence of PDC (*PDC alone*) remained independently significant. The AUC was 0.808 (95% CI 0.717–0.880) for *TB* > 1 + *PDC* and 0.79 (95% CI 0.699–0.866) for *PDC alone*, both sets were statistically significant (*p* = 0.001). Sensitivity and specificity were 68.7% and 92.8%, and 75% and 84.52%, respectively. The Akaike information criterion was 64.129 for *TB* > 1 + *PDC* and 71.012 for *PDC alone*, confirming the better quality of *TB* > 1 + *PDC* criteria.

**Figure 5:**
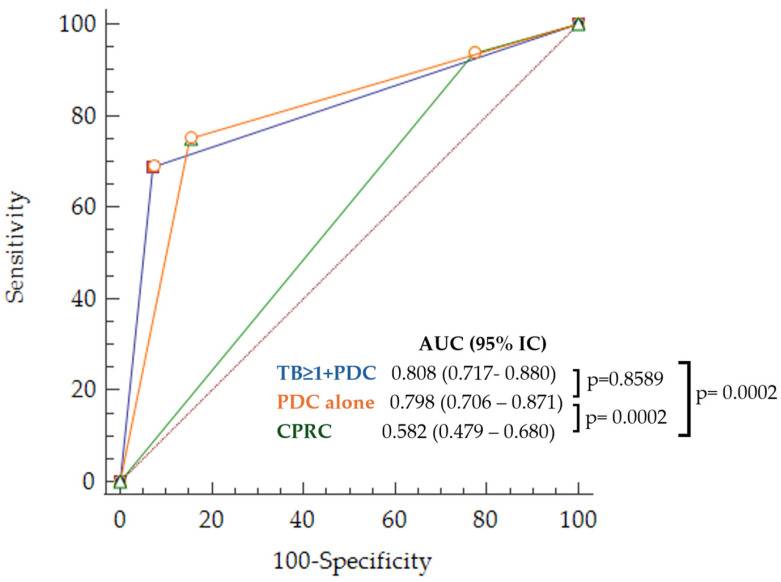
ROC curves for LNM prediction corresponding to the obtained sets of risk criteria and common practice (*TB* > 1 + *PDC* in blue, *PDC alone* in orange, and CPRC in green).

Both sets of criteria would significantly reduce the proportion of false positives, and, therefore, unnecessary surgeries, by 41.5% for *TB* > 1 + *PDC* and by 34.7% for *PDC alone*, without increasing the proportion of false negatives (patients with LNM who would not be offered additional surgery). Figure 5 and Table 2 show the ROC curves for the parameters related to the predictive capacity and quality of each of the two sets, as well as for the CPRC considering a prevalence of 16% for LNM in the sample. Differences in false positives and negatives are shown in Table 3.

## 6. Association with Poor Outcome

The association analysis with the variable ‘poor outcome’, defined by the presence of LNM in the locoregional surgical resection specimen and/or synchronous distant metastases, or the occurrence of metastasis or local recurrence during follow-up, was conducted in 161 cases: 100 patients treated with locoregional surgery and 61 with local resection alone with at least two years’ follow-up (Table 4). The median follow-up of the patients treated exclusively with local resection was 72 months (19–289). There were 23 cases with poor outcome (14.28%): 16 with LNM included in the association with LNM analysis, 1 with synchronous hepatic metastases, and 6 with adenocarcinoma recurrence (2 locally and 4 distant). The same approach as in the analysis of association with LNM was followed.

**Figure 6:**
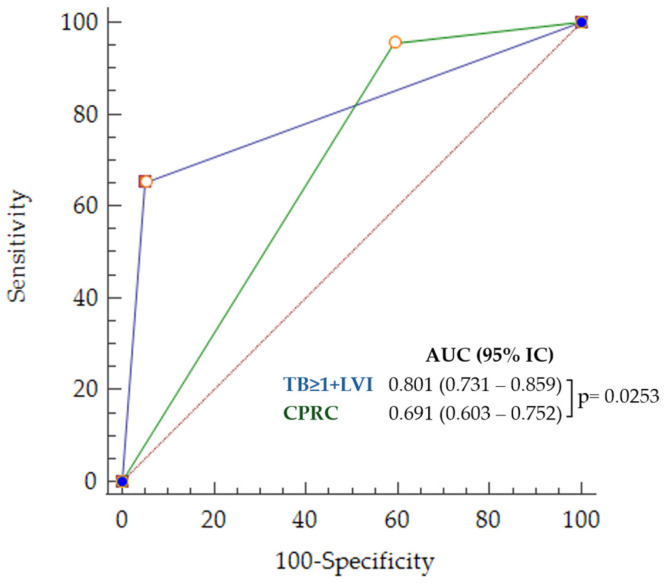
ROC curves for poor outcome prediction corresponding to *LVI* + *TB* > 1 (in blue) and CPRC (in green).

In the multivariate analysis, only LVI and the presence of TB > 1 emerged as independent variables (*LVI* + *TB* > 1), regardless of the variable selection procedure. The ROC curves and corresponding diagnostic parameters for this set of criteria and routine clinical practice are displayed in Figure 6 and Table 5.

A statistically significant (*p* < 0.0001) decrease of 45% (CI 95% = 22.8–63.1%) in false positives was observed using only LVI and TB > 1 compared to CPRC. In contrast, the 3.1% (CI 95% −4.5–8.0%) increase in the proportion of false negatives was not statistically significant (*p* = 0.28).

## 7. Discussion

Our study suggests that eliminating quantitative variables, such as depth of submucosal invasion from CPRC could reduce the number of patients undergoing unnecessary surgeries by approximately 40% (34.7–45%) without increasing the number of untreated LNM. In the analysis focusing exclusively on predicting the risk of locoregional nodal involvement, only TB > 1 and PDC emerged as independent risk factors. However, in the analysis that also considered the possibility of local recurrence and synchronous or metachronous metastases, (‘poor outcome’), the independent risk variables were LVI and TB > 1. We do not believe that there is a contradiction between the results of the two analyses because TB and PDC are both morphological expressions of an EMT (as is also the immature desmoplastic stroma [23], which was not evaluated in this study), and the predominance of one over the other may simply occur by chance. EMT is a process through which tumor cells lose their epithelial properties and acquire the capacity of mesenchymal cells to migrate through the extracellular matrix, invade blood vessels, and metastasize. EMT plays a crucial role in cancer progression and is a significant area of research in oncology [24,25]. The fact that in the first analysis we found an association between LVI and LNM, but not as an independent risk factor, is likely due to the smaller sample size of the sub-study, as LVI is a well-documented risk factor with a high capacity to predict the presence of LNM consistently observed in multiple studies [26,27,28,29,30,31,32,33,34,35].

Neither histological grade, nor depth of submucosal invasion, nor margin involvement, all variables used in decision-making in routine clinical practice, emerged as independent risk factors in any of the sub-studies. The differentiation grade, whether classified in two or more categories (histological grade), is known to correlate with the presence of LNM and prognosis. Differentiation grade assessment is traditionally based on the proportions of well or poorly differentiated components. This definition is somewhat subjective and entails a considerable interobserver variability [36,37,38]. Thus, some guidelines, including European recommendations, suggest classifying tumors with any amount of poorly differentiated components as high-grade tumors [22]. In fact, in our study, G2 tumors (and, therefore, with low histological grade) were significantly associated with LNM and ‘poor outcome’. Moreover, G2 and even G1 tumors with PDC were significantly associated with LNM or ‘poor outcome’, while G1 and G2 tumors without PDC were not. These findings support the superiority of the prognostic value of PDC in T1 CRC compared to conventional differentiation grade and, as suggested by other authors, PDC may be more reliable than the traditional system for tumor grading [10,11,12,13,14,15,16,17,39,40].

Deep submucosal invasion was statistically significant only in the univariate analysis of association with poor outcome, but not in the multivariate analysis. The association between the depth of invasion and the presence of lymph node metastasis has been well documented across multiple studies, although not as consistently as the variables mentioned earlier. Several studies with a large sample size, including a recent metanalysis [26,41,42], confirm that, on its own, depth of invasion is not an independent risk factor. Furthermore, the 1000 µm cutoff point, used to decide on the indication of radical surgery after endoscopic resection in the last 20 years, has a low positive predictive value, leading to a high number of unnecessary laparotomies [30].

The presence of a positive deep margin was not associated with lymph node metastasis or poor outcome. Therefore, we believe that this factor alone should not indicate ALRS. It could justify an extension of the local resection to ensure complete tumor removal, a procedure that can now be performed in most cases without the need for surgery, thanks to the development of full-thickness endoscopic resection [43]. Variables that require measurement, such as depth of invasion and margin status, also present the drawback that their assessment is often impossible in piecemeal resections. In contrast, LVI, TB, and PDC can be evaluated in fragmented specimens. This is a significant advantage because, while it is ideal for endoscopists to perform en-bloc resection when suspecting invasive carcinoma, it is inevitable that pathologists will receive a considerable number of fragmented samples (16% in our series). In these cases, there is a tendency to assume deep invasion and affected margin to avoid leaving untreated tumors, thereby contributing to the high number of unnecessary additional surgeries. This is especially relevant considering that these variables have a much weaker association compared to LVI and EMT expressions.

The role of tumor location in T1 CRC is controversial. Most of the studies show an association between rectal and/or sigmoid location and LNM [44,45,46], but this factor has not been included in practice guidelines. In our investigation, rectal lesions were associated with both LNM and ‘poor outcome’ in the univariate analysis, but not as an independent factor, so our results do not support additional surgery when the only risk factor is rectal location.

One could argue that the magnitude of the difference in the proportion of false positives observed between the sets of criteria obtained and the common practice criteria might be exaggerated because, in real routine clinical practice, the risk criteria are applied on an individualized basis, and not all variables are given the same weight in every case. For instance, in cases of lower rectal location, where additional surgery carries a high risk of functional sequelae, the application of certain criteria may be more flexible, or nonsurgical alternatives such as adjuvant chemotherapy or radiotherapy might be offered instead of total mesorectal excision with sphincter amputation. However, in our series, only 8 out of the 51 patients (15.7%) who underwent additional surgery after local resection had lymph node metastasis; in other words, 84.3% were false positives where no metastases were found after the additional surgery, which is very similar to the 86.7% predicted with a strict application of the criteria and to those demonstrated in other studies [15,47,48,49]. In the management of T1 CRC, it is not only important to try to reduce false positives but also to decrease, or at the very least maintain, the low rates of false negatives observed with standard criteria. It is worth noting that two of the six recurrence cases occurred in patients treated exclusively with local resection in whom LVI and TB > 1 had gone unnoticed and were only identified upon a thorough review of the slides for the study. This underscores the importance of a very careful review of T1 colorectal cancer cases. This observation aligns with that of Kim et al., who point out that a substantial portion of cases not undergoing additional surgery and experiencing poor outcomes are misclassified into the low-risk group due to failure to identify adverse prognostic factors [50].

The fundamental limitation of our study is the small sample size, which prevented a more decisive assertion regarding the superiority of LVI and EMT expressions over routinely employed criteria. In the particular issue of false negatives, in spite of the fact that differences with CPRC were not statistically significant, the confidence intervals are not narrow enough to discard the possibility of a clinically relevant increase in the proportion of patients with LNM who are not offered additional surgery. Nevertheless, we have been able to verify the role of PDC, the variable from which the sample size calculation was performed, which so far has been less studied than TB. Several recent studies focus on identifying a group of patients with a very low risk, in whom surgery can be avoided with the almost complete certainty of no nodal involvement. Specifically, Morini et al. observe in their series that patients with well-differentiated tumors (G1), no lymphovascular invasion, and an invasive front width <4.25 mm, can be safely followed without additional surgery [51]. Moreover, Ozeki et al. identify a subset of patients without lymph node metastases and no recurrences, characterized by the presence of a well differentiated adenocarcinoma, absence of LVI, a negative vertical margin, a depth of less than 2000 µm, and colonic location [52]. However, they limit their conclusions to cases where en-bloc resection has been carried out, which significantly restricts the number of patients who can avoid additional surgery. Kim et al. propose a scoring system where patients with a score of 0 (well or moderately differentiated tumors, absence of LVI, and TB grade < 2) have a risk of lymph node metastasis of 5.6% [53]. In contrast, Piao et al. find that in patients without poor differentiation and without LVI, the risk is only 0.8% [54]. The criteria described in these last two studies identify low-risk patient groups without using quantitative variables, as do the criteria proposed in our study. Our criteria, unlike those of Kim et al. and Piao et al., do not include differentiation grade, which could be an advantage, given the mentioned limitations of conventional grading and the variability in its definition.

The development of a method capable of directly identifying, without estimations, the presence or absence of lymph node metastasis in colorectal cancer would allow a clear distinction between patients who would benefit from additional surgery and those for whom local treatment is sufficient. Radiomics, which transforms fundamentally qualitative data generated from imaging tests into large amounts of quantitative data and processes and integrates them using artificial intelligence, may possibly facilitate advances in non-invasive diagnosis in the coming years [55,56,57,58,59,60]. On the other hand, advances in molecular biology could contribute significantly to the management of T1 CRC: a model recently created by the Miyazaki group [61], which combines a panel of exosomal and free miRNAs in serum along with key histological data, could reduce unnecessary surgeries by 76% compared to traditional criteria, with less than 1% of cases having unidentified nodal involvement. While these authors acknowledge limitations due to a small sample size and inclusion of only patients with Japanese ancestry, the results are very promising.

## 8. Conclusions

Until reliable non-invasive methods for diagnosing nodal status are developed, histological factors will undoubtedly remain the cornerstone of early colorectal carcinoma management for several years to come, with or without the support of artificial intelligence and molecular biology. Decision criteria based solely on the presence of LVI and on expressions of EMT, such as TB > 1 and PDC, could allow for a reduction in unnecessary surgeries by more than one third of the cases without increasing the proportion of patients with nodal involvement who are not offered additional surgery, provided that a thorough pathologic assessment is carried out.

## Figures and Tables

**Table 1:** Association of the different risk factors with LNM. Univariate analysis.

Variable	LNM−	LNM+	OR (CI 95%)	*p*
Sex				
- Female	44 (81.5%)	10 (18.5%)		
- Male	40 (87%)	6 (13%)	0.66 (0.22–1.98)	0.4587
Age (median)	66	59	0.92 (0.871–0.99)	**0.024**
Location				
- Colon	71 (88.7%)	9 (11.3%)		
- Rectum	13 (65%)	7 (35%)	4.24 (1.34–13.43)	**0.0138**
Median endoscopically estimated size in mm	30	30	1.00 (0.96–1.04)	0.9333
(range)	(6–80)	(10–50)		
Median histologically measured size in mm	18	18	0.98 (0.93–1.04)	0.5882
(range)	(5–65)	(12–44)		
Morphology				
Pedunculated	6 (85.7%)	1 (14.3%)		
Non-pedunculated	78 (83.9%)	15 (16.1%)	--	0.8984
Lymphovascular invasion				
Absent	72 (93.5%)	5 (6.5%)		
Present	12 (52.2%)	11 (47.8%)	17.25 (6.09–48.82)	**<0.0001**
Depth of invasion				
<1000 µm	23 (92%)	2(8%)	Ref.	
≥1000 µm	49 (79%)	13 (21%)	3.05 (0.635–14.650)	0.1635
Not evaluable	12 (92.3%)	1 (7.7%)	0.95 (0.07–11.67)	0.9734
Differentiation grade				
- G1	73 (91.2%)	7(8.8%)	Ref.	
- G2	9 (60%)	6(40%)	6.95 (1.91–2 5.29)	**0.0033**
- G3	2 (40%)	3 (60%)	15.64 (2.22–109.95)	**0.0057**
- G4	0	0	--	--
Histologic grade				
G1 or G2	82 (86.3%)	13(13.7%)	Ref.	
G3 or G4	2 (40%)	3 (60%)	9.46 (1.44–62.15)	**0.0193**
TB grade				
1	78 (94%)	5 (6%)	Ref.	
2	4 (44.4%)	5 (55.6%)	19.50 (3.95–96.1)	**0.0003**
3	2 (25%)	6 (75%)	46.80 (7.44–294.12)	**<0.0001**
PDC				
Absent	70 (94.6%)	4 (5.4%)	Ref.	
Present	14 (53.8%)	12 (46.2%)	15.00 (4.21–53.34)	**<0.0001**
Differentiation grade classified by PDC				
G1 PDC−	64(95.5%)	3 (4.5%)	Ref.	--
G1 PDC+	9 (70%)	4 (30%)	9.48 (1.81–40.44)	**0.0076**
G2 PDC−	6 (86%)	1 (14%)	3.55 (0.31–39.7)	0.3028
G2 PDC+	3 (38%)	5(62%)	35.55 (5.64–224.10)	**0.0001**
G3 PDC−	0 (0%)	0 (0%)	--	--
G3 PDC+	2 (40%)	3 (60%)	32.0(3.79–269.59)	**0.0014**
*Muscularis mucosae* disruption				
Incomplete	65 (86.7%)	10 (13.3%)	Ref.	0.0667
Complete	13 (68.4%)	6 (31.6%)	3.00 (0.92–9.70)	
Not evaluable	6 (100%)	0 (0%)	--	
Width of invasion				
<4000 µm	64(84.2%)	12 (15.8%)	Ref.	
≥4000 µm	5 (64.5%)	3 (37.5%)	3.2 (0.67–15.20)	0.1435
Not evaluable	15 (93.7%)	1 (6.2%)	0.35 (0.04–2.95)	0.3382
Intratumoral inflammation				
Absent or mild	58 (81.7%)	13 (18.3%)	Ref.	
Moderate/intense	26 (89.7%)	3 (10.3%)	0.51 (0.13–1.96)	0.3307
Peritumoral inflammation				
Absent or mild	34 (85%)	6 (15%)		
Moderate/intense	50 (83.3%)	10 (16.7%)	1.13 (0.37–3.41)	0.8238
Background adenoma				
Present	81 (84.4%)	15 (15.6%)	Ref.	
Absent	3 (75%)	1 (25%)	1.80 (0.17–18.48)	0.6209
Macroscopically complete resection				
- Yes	81 (83.3%)	16(16.7%)		
- No	3 (100%)	0 (0%)	--	--
Resection margin				
- >1 mm	48 (84.2%)	9 (15.8%)	Ref.	
- ≤1 mm	28 (82.4%)	6 (17.65)	1.88 (0.22–16.06)	0.5638
- Not evaluable	8 (88.9%)	1 (11.1%)	1.12 (0.06–21.08)	0.9372
En bloc resection				
- Yes	68 (82.9%)	14 (17.1%)	Ref.	
- No	16 (88.9%)	2 (11.1%)	0.56 (0.10–3.12)	0.511
Residual neoplasia after endoscopic resection (in surgical specimen)				
- No	41 (83.7%)	8 (16.3%)		
- Yes	2 (100%)	0 (0%)	--	--
- Not applicable	41 (83.7%)	8 (16.3%)		

Ref: reference; OR: odds ratio; CI: confidence interval; TB: tumor budding; PDC: poorly differentiated clusters. Statistically significant *p*-values in bold.

**Table 2:** Diagnostic values for LNM prediction of the obtained sets of risk criteria and common practice in percentages.

	SE	SP	PPV	NPV	FP	FN	AIC
TB > 1 + PDC	68.7	92.8	54.8	95.9	45.2	4.1	64.129
PDC alone	75	84.52	48	94.7	52	5.3	71.012
CPRC	93.75	22.72	13.3	96.6	86.7	3.4	--

SE: sensitivity; SP: specificity; PPV: positive predictive value; NPV: negative predictive value; FP: false positives; FN: false negatives; AIC: Akaike information criterion.

**Table 3:** Differences in false positives (FP) and false negatives (FN) between the obtained sets of risk criteria and common practice.

	TB > 1 + PDC vs. PDC Alone		TB > 1 + PDC vs. CPRC		PDC Alone vs. CPRC	
FP	6.8 (−22.1–34.1)	*p* = 0.67	41.5 (17.4–62.8)	*p* = 0.0001	34.7 (14.5–54.1)	*p* = 0.0002
FN	0.7 (−17.4–7.9)	*p* = 088	1.2 (−6.2–9.7)	*p* = 0.72	1.9 (−16.3–10.03)	*p* = 0.73

**Table 4:** Association of the different risk factors with poor outcome. Univariate analysis.

Variable	No Poor Outcome	Poor Outcome	OR (CI 95%)	*p*
Sex				
- Female	66 (84.6%)	12 (15.4%)		
- Male	72 (86.7%)	11 (13.3%)	1.19 (0.49–2.87)	0.6995
Age (median)	66	59	0.92 (0.87–0.99)	**0.024**
Location				
- Colon	109 (89.3%)	13 (10.7%)	Ref.	
- Rectum	29 (74.4%)	10 (25.6%)	2.891 (1.15–7.25)	**0.0238**
Median endoscopically estimated size in mm	30	30	1.00 (0.97–1.09)	0.5677
(range)	(6–80)	(10–50)		
Median histologically measured size in mm	18	16.97	0.98 (0.93–1.03)	0.4327
(range)	(5–65)	(7–44)		
Morphology				
Pedunculated	31 (91.2%)	3 (8.8%)	Ref.	
Non-pedunculated	107 (84.3%)	20 (15.7%)	0.51 (0.14–1.85)	0.3126
Lymphovascular invasion				
Absent	127 (93.4%)	9 (6.6%)	Ref.	
Present	11 (44%)	14 (56%)	17.95 (6.35–50.79)	**<0.0001**
Depth of invasion				
<1000 µm	60 (95.2%)	3 (4.8%)	Ref.	
≥1000 µm	64 (79%)	17 (21%)	5.31 (1.48–19.04)	**0.0104**
Not evaluable	14 (82.4%)	3 (17.6%)	4.28 (0.78–23.52)	0.0939
Differentiation grade				
- G1	120(90.9%)	12(9.1%)	Ref.	
- G2	17 (70.8%)	7(29.2%)	4.117 (1.424–11.903)	**0.009**
- G3	1(20%)	4 (80%)	40 (4.131–387.282)	**0.0014**
- G4	0	0	--	--
Histologic grade				
G1 or G2	137 (87.8%)	19(12.2%)	Ref.	
G3 or G4	1 (20%)	4 (80%)	28.842 (3.060–271.809)	**0.0033**
TB grade				
1	131 (94%)	8 (6%)	Ref.	
>1	7 (44.4%)	15 (55.6%)	5.923 (3.339–10.508)	**<0.0001**
PDC				
Absent	120 (93.7%)	8 (6.3%)	Ref.	
Present	18 (54.5%)	15 (45.5%)	12.500 (4.640–33.668)	**<0.0001**
Differentiation grade classified by PDC				
G1 PDC−	109 (94%)	7 (6%)	Ref.	
G1 PDC+	11 (68.7%)	5 (31.2%)	7.07 (1.92–26.08)	**0.0033**
G2 PDC−	6 (90.1%)	1 (9.1%)	1.55 (0.17–13.95)	0.6923
G2 PDC+	7(53.8%)	5(46.2%)	13.34 (3.52–50.54)	**0.0001**
G3 PDC−	0 (0%)	0 (0%)	--	--
G3 PDC+	1 (20%)	4(80%)	62.28 (6.11–634.29)	**0.0005**
*Muscularis mucosae* disruption				
Incomplete	112 (88.2%)	15 (11.8%)	Ref.	
Complete	16 (69.6%)	7 (30.4%)	3.26 (1.1559–9.2319)	**0.0255**
Not evaluable	10 (90.9%)	1 (9.1%)	0.74 (0.09–6.25)	0.7876
Width of invasion				
<4000 µm	109 (87.9%)	15 (12.1%)	Ref.	
≥4000 µm	6 (54.5%)	5 (45.5%)	6.05 (1.64–22.30)	**0.0068**
Not evaluable	23 (88.5%)	3 (11.5%)	0.94 (0.25–3.54)	0.9365
Intratumoral inflammation				
Absent or mild	101 (83.5%)	20 (16.5%)	Ref.	
Moderate/intense	36 (92.3%)	3 (7.7%)	0.42 (0.12–1.51)	0.1872
Peritumoral inflammation				
Absent or mild	54 (84.4%)	10 (15.6%)	Ref.	
Moderate/intense	83 (86.5%)	13 (13.5%)	1.19(0.49–2.92)	0.6935
Background adenoma				
Present	135 (86%)	22 (14%)		
Absent	3 (75%)	1 (25%)	0.48 (0.04–4.91)	0.5433
Macroscopically complete resection				
- Yes	127(83.3%)	23(16.7%)		
- No	5 (100%)	0 (0%)	--	--
Resection margin				
- >1 mm	73 (81.1%)	17 (8.9%)	Ref.	
- ≤1 mm	50 (92.6%)	4 (7.4%)	2.91 (0.92–9.16)	0.0679
- Not evaluable	9 (81.8%)	2 (8.2%)	2.77 (0.44–17.48)	0.2764
En bloc resection				
- Yes	110 (85.3%)	19 (14.7%)	Ref.	
- No	22 (84.6%)	4 (15.4%)	1.05 (0.32–3.39)	0.9316
Residual neoplasia after endoscopic resection (in surgical specimen)				
- No	95 (86.4%)	15 (15.6%)		
- Yes	2 (100%)	0 (0%)	--	--
- Not applicable	41 (83.7%)	8 (16.3%)		

Ref: reference; OR: odds ratio; CI: confidence interval; TB: tumor budding; PDC: poorly differentiated clusters. Statistically significant *p*-values in bold.

**Table 5:** Diagnostic values for adverse outcome prediction of LVI + TB > 1 and CPRC.

	SE	SP	PPV	NPV	FP	FN
LVI + TB > 1	65.2	94.9	61.9	95.6	38.1	4.4
CPRC	95.6	40.6	16.9	98.7	83.1	1.3

SE: sensitivity; SP: specificity; PPV: positive predictive value; NPV: negative predictive value; FP: false positives; FN: false negatives.

## Data Availability

The data presented in this study are available on request from the corresponding author.
